# Supplementary material for: A benzochalcone derivative synchronously induces apoptosis and ferroptosis in pancreatic cancer cells
Source: PeerJ. 2023 Nov 1;11:e16291. doi: 10.7717/peerj.16291 (PMC10625348; doi:10.7717/peerj.16291)
Supplement: Supplemental Information 1 [file peerj-11-16291-s001.docx]

Supplemental file 1. Uncropped pictures of western blot

PANC-1 Mia PACA2

KL-6 (μΜ) 0 0.5 1 2 0 0.5 1 2


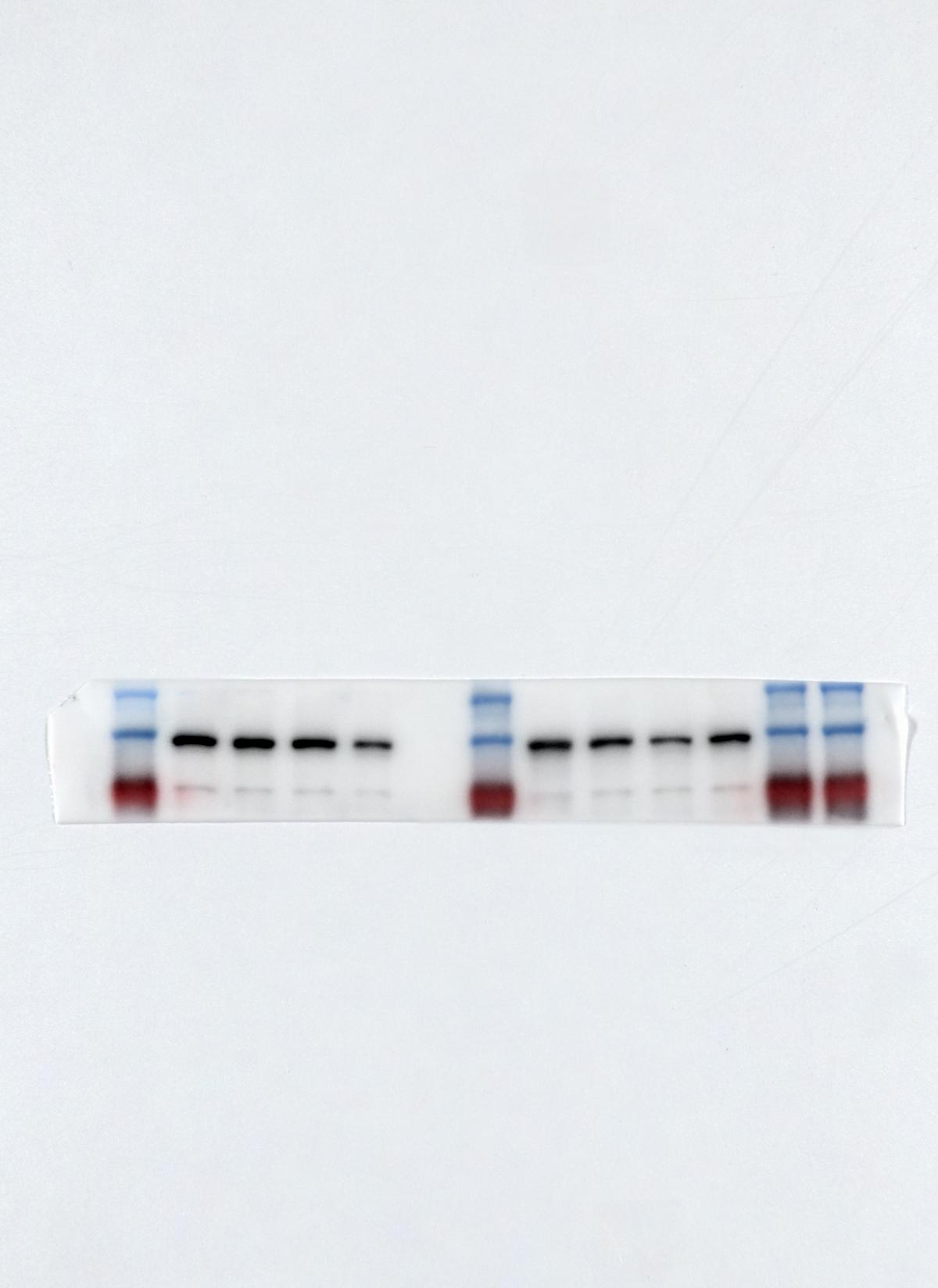
 DDX27


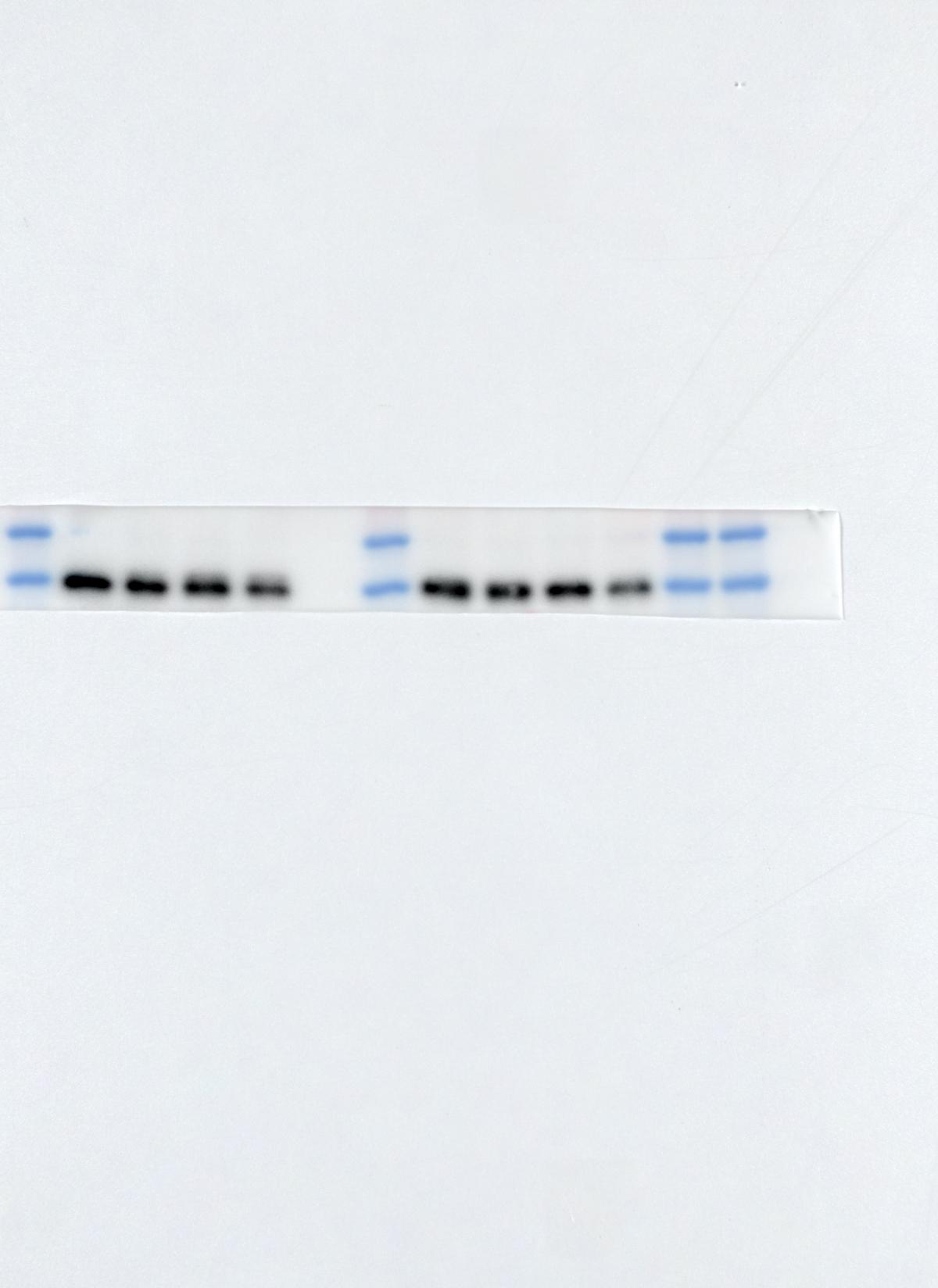
 MCL1


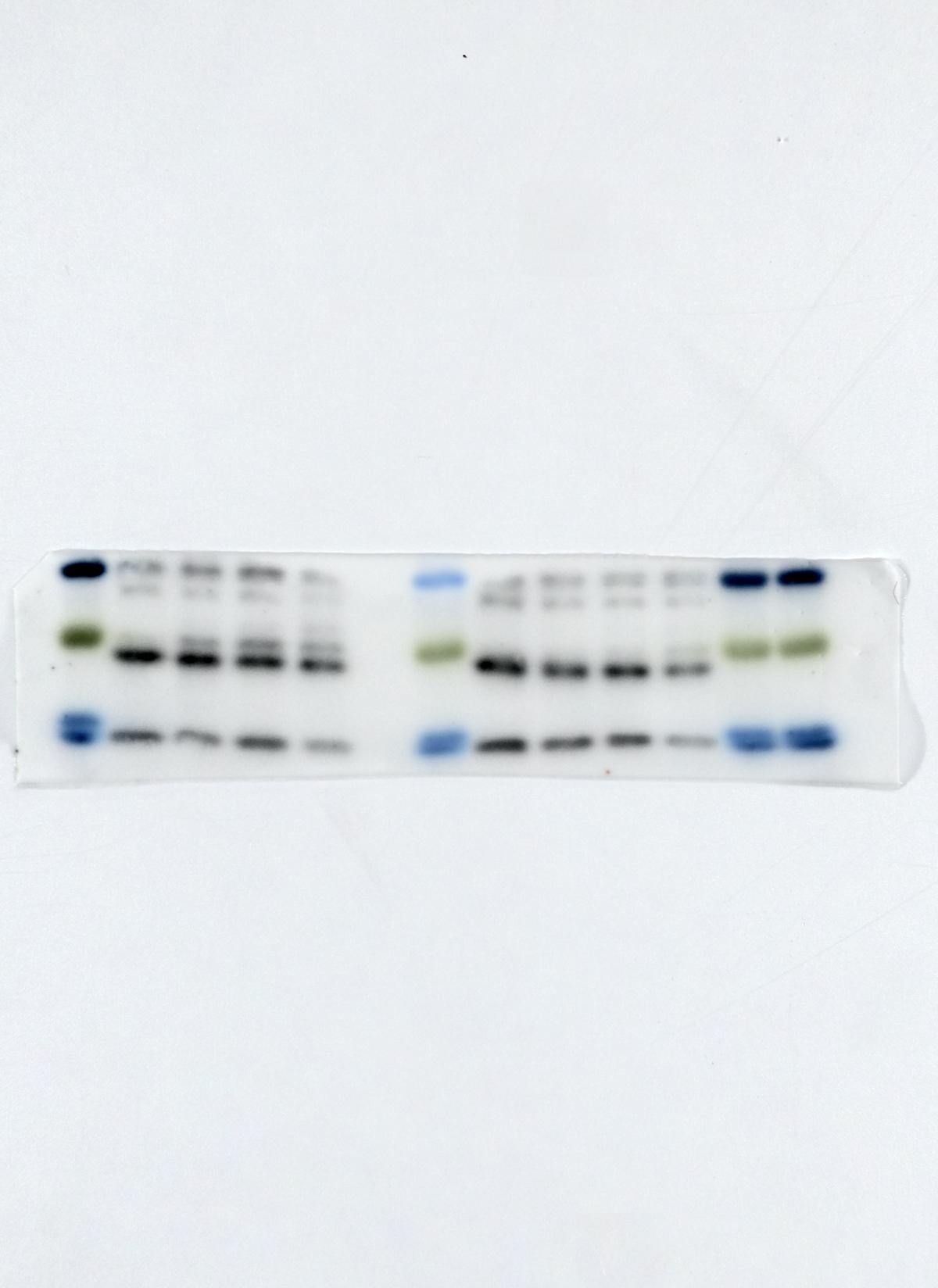
 Bcl-2


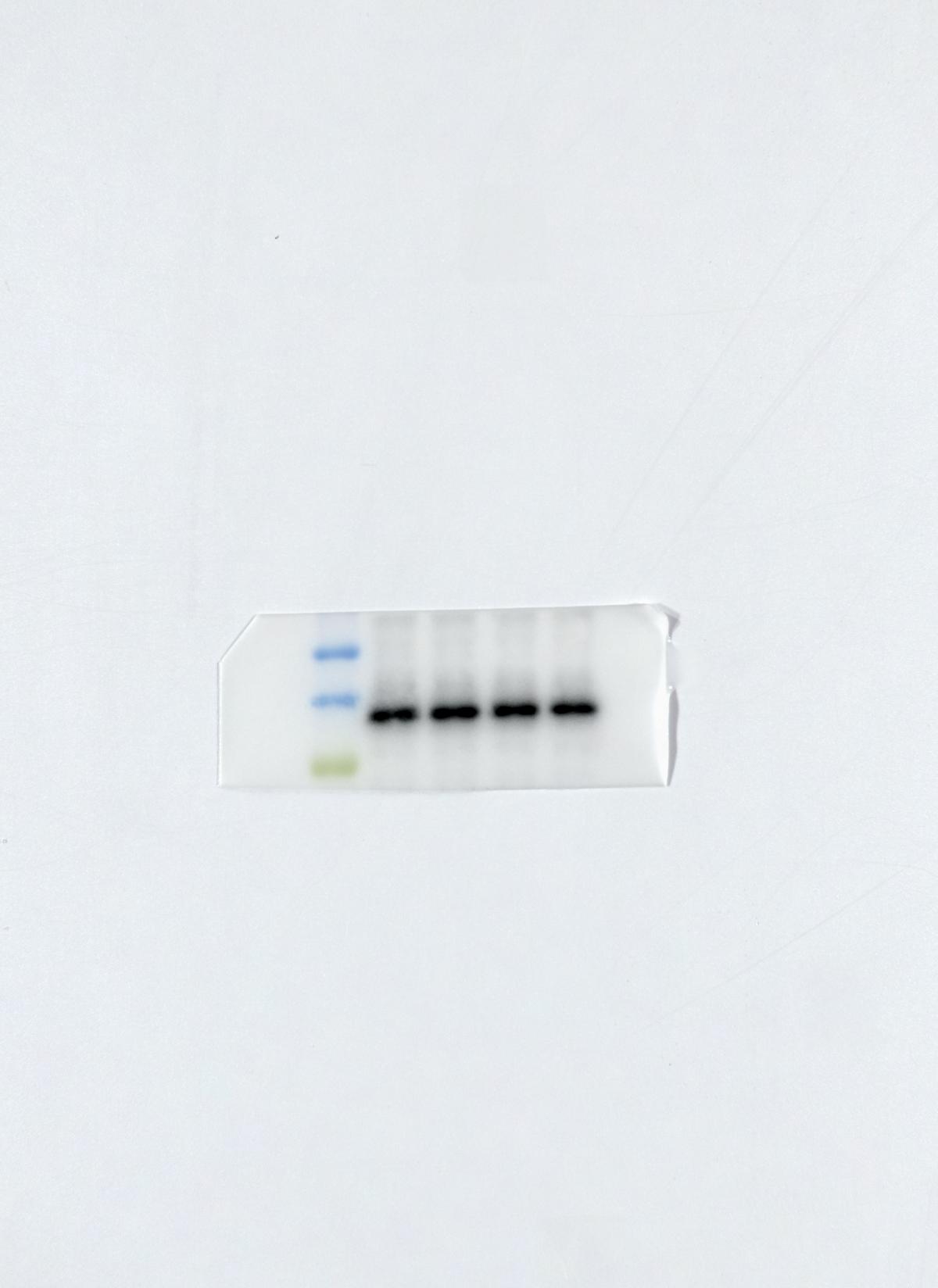

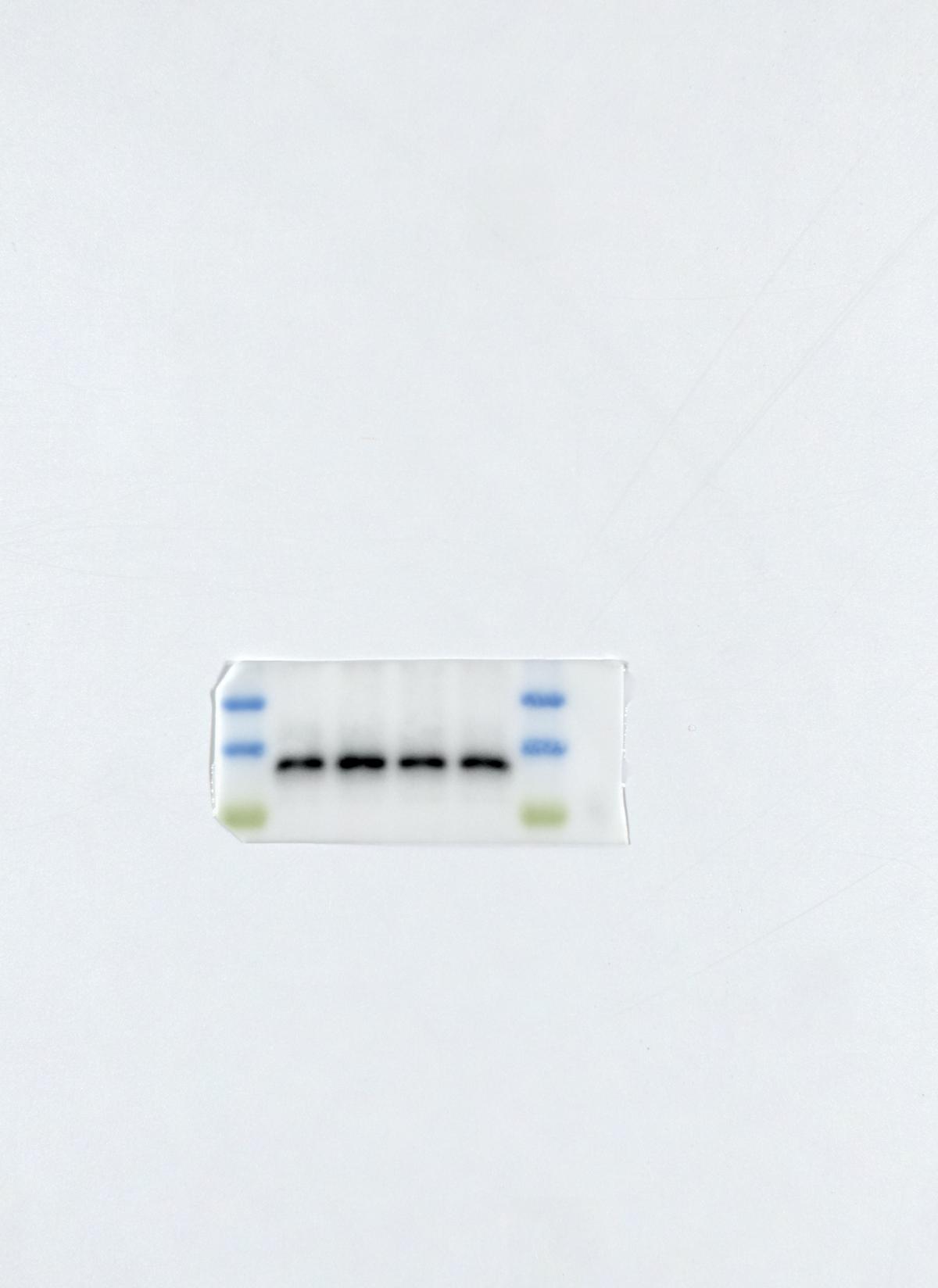
 Caspase3


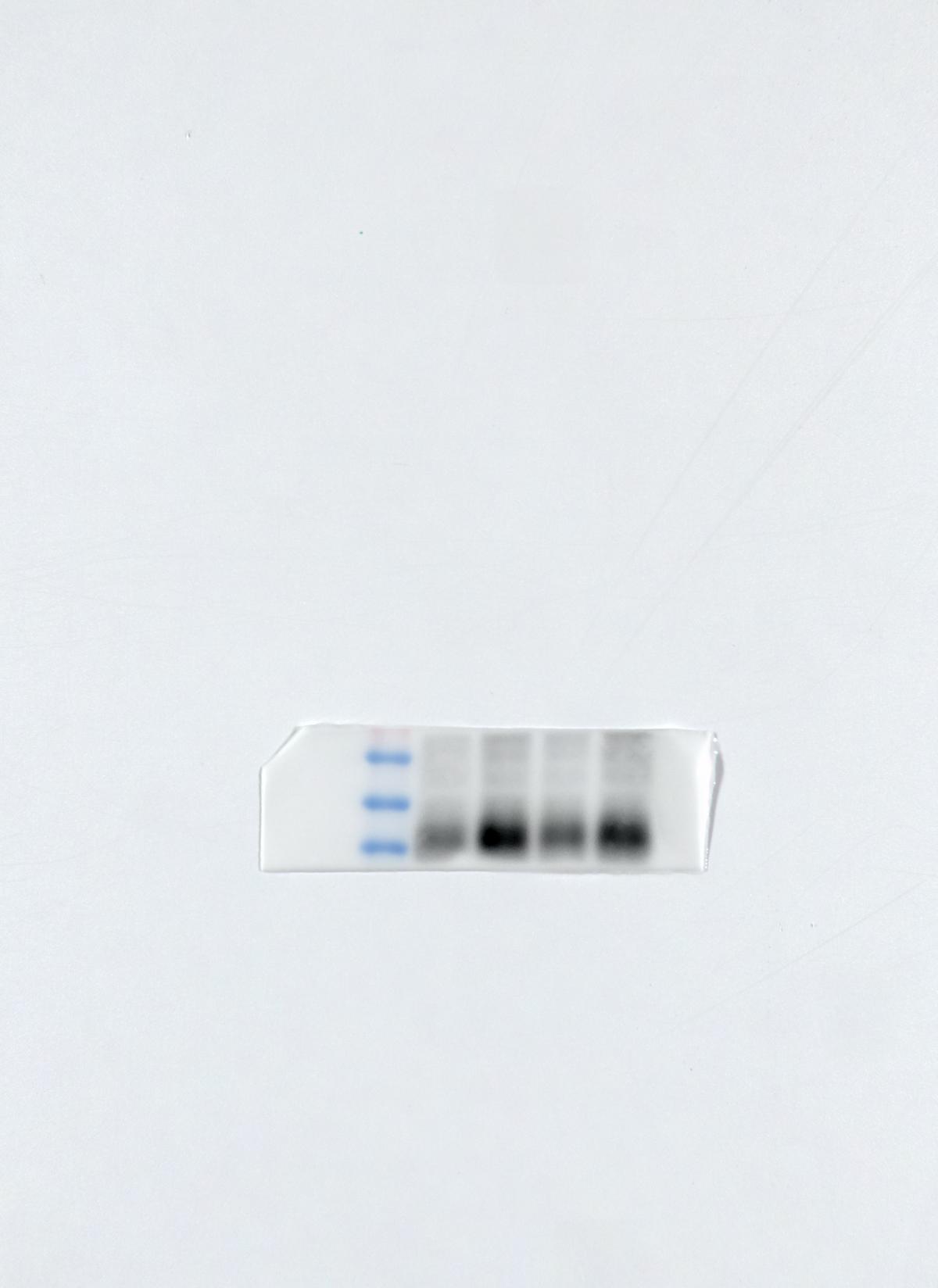

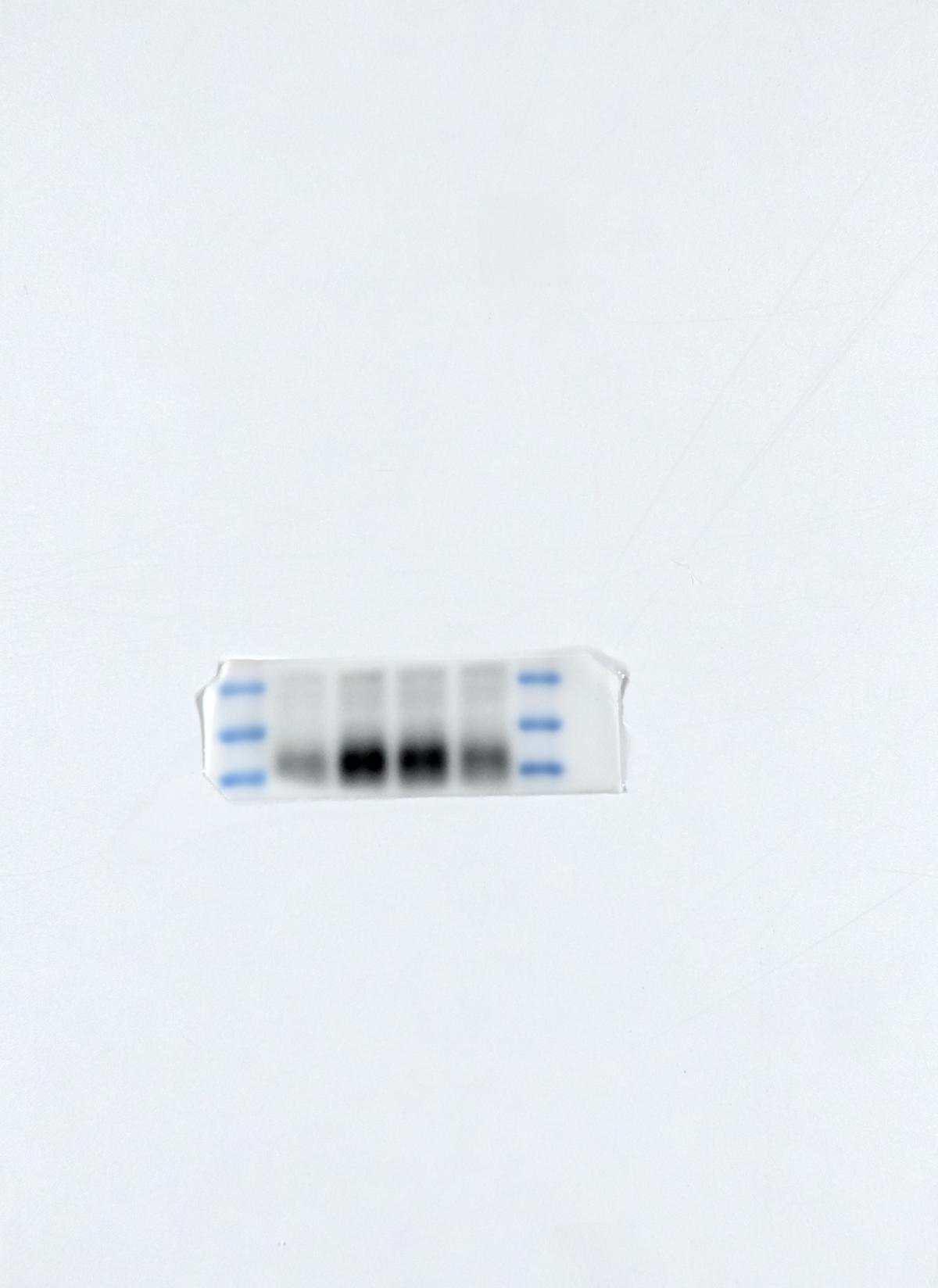
 SLC7A11


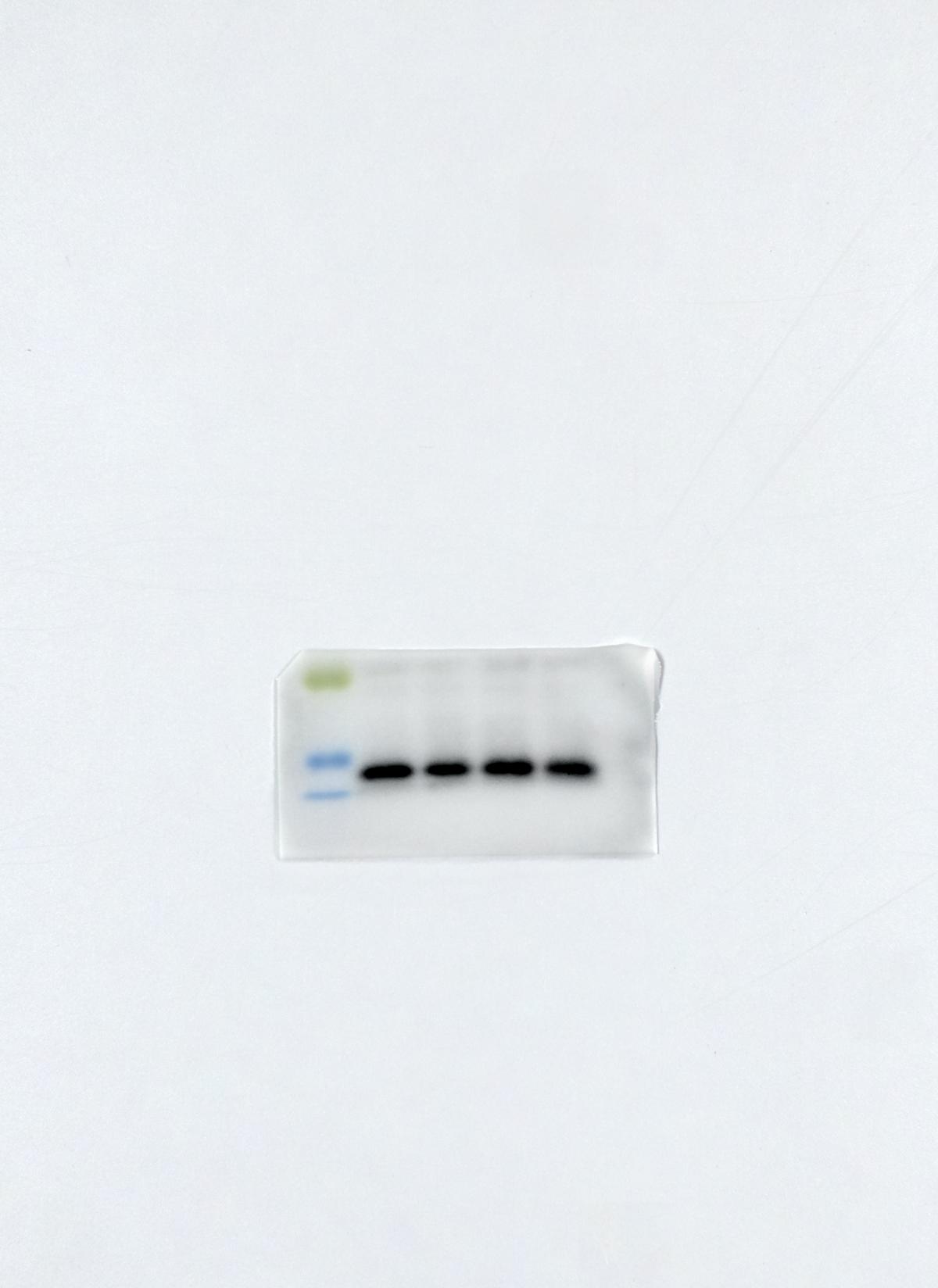

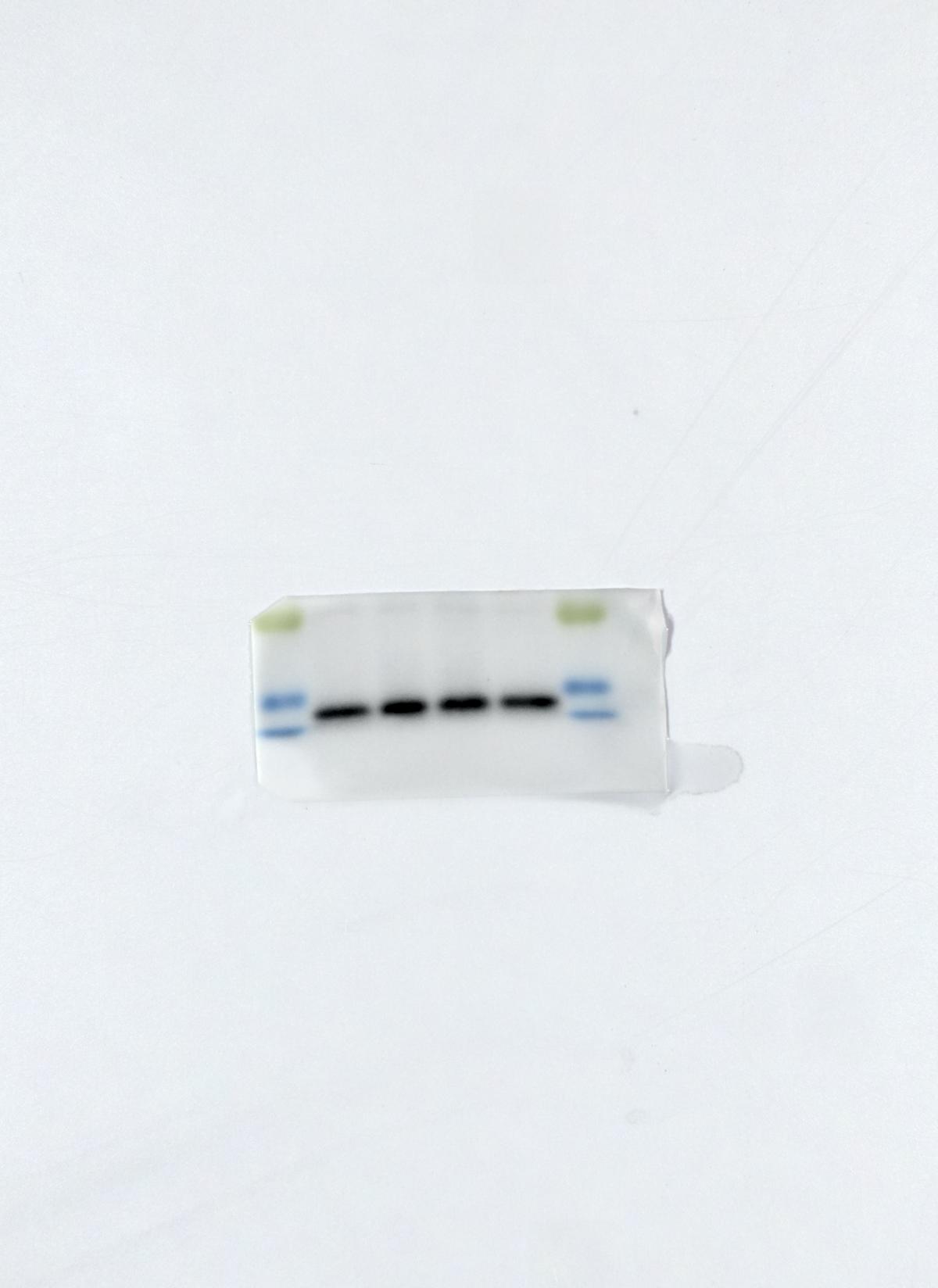
 GPX4


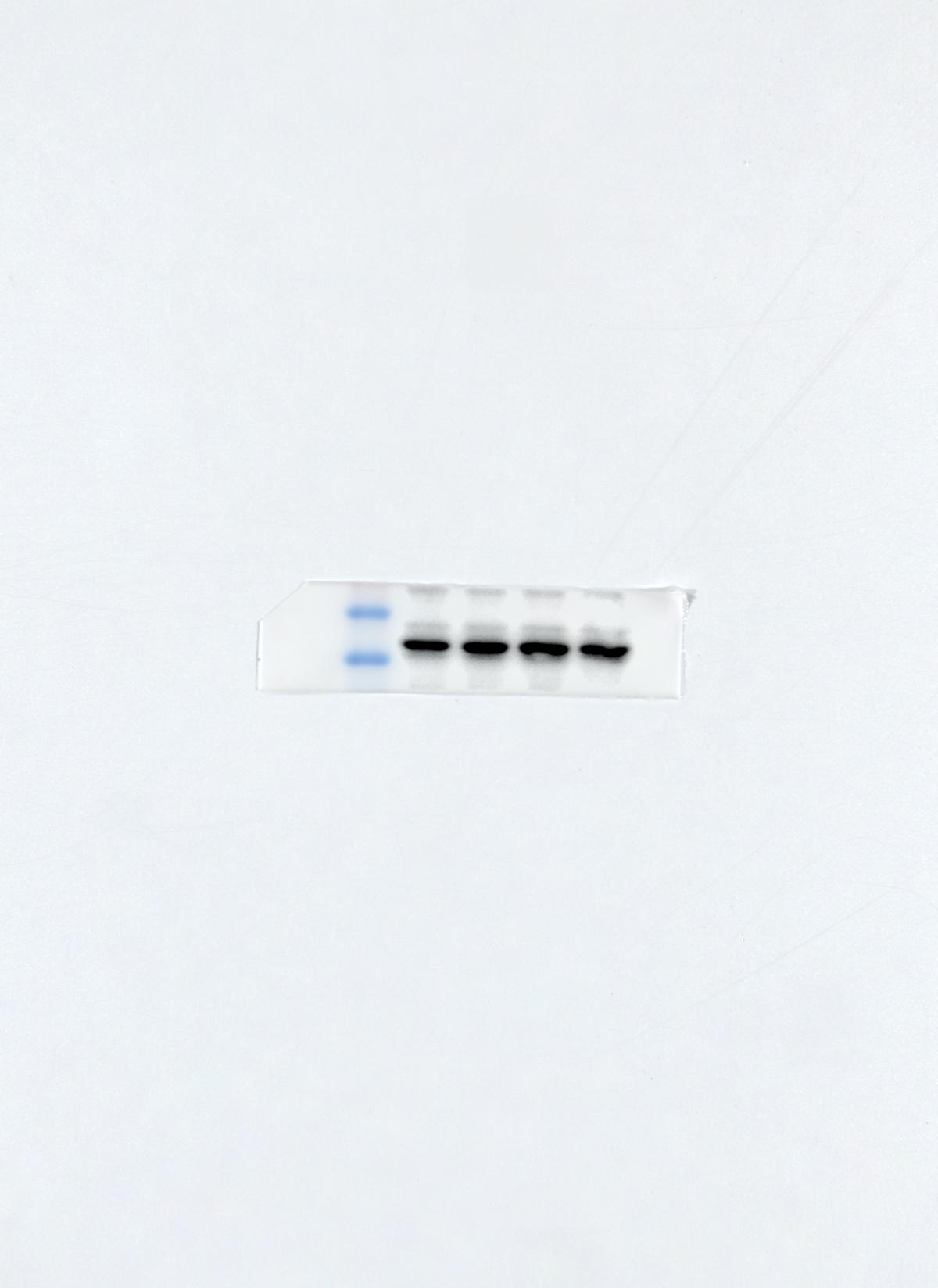

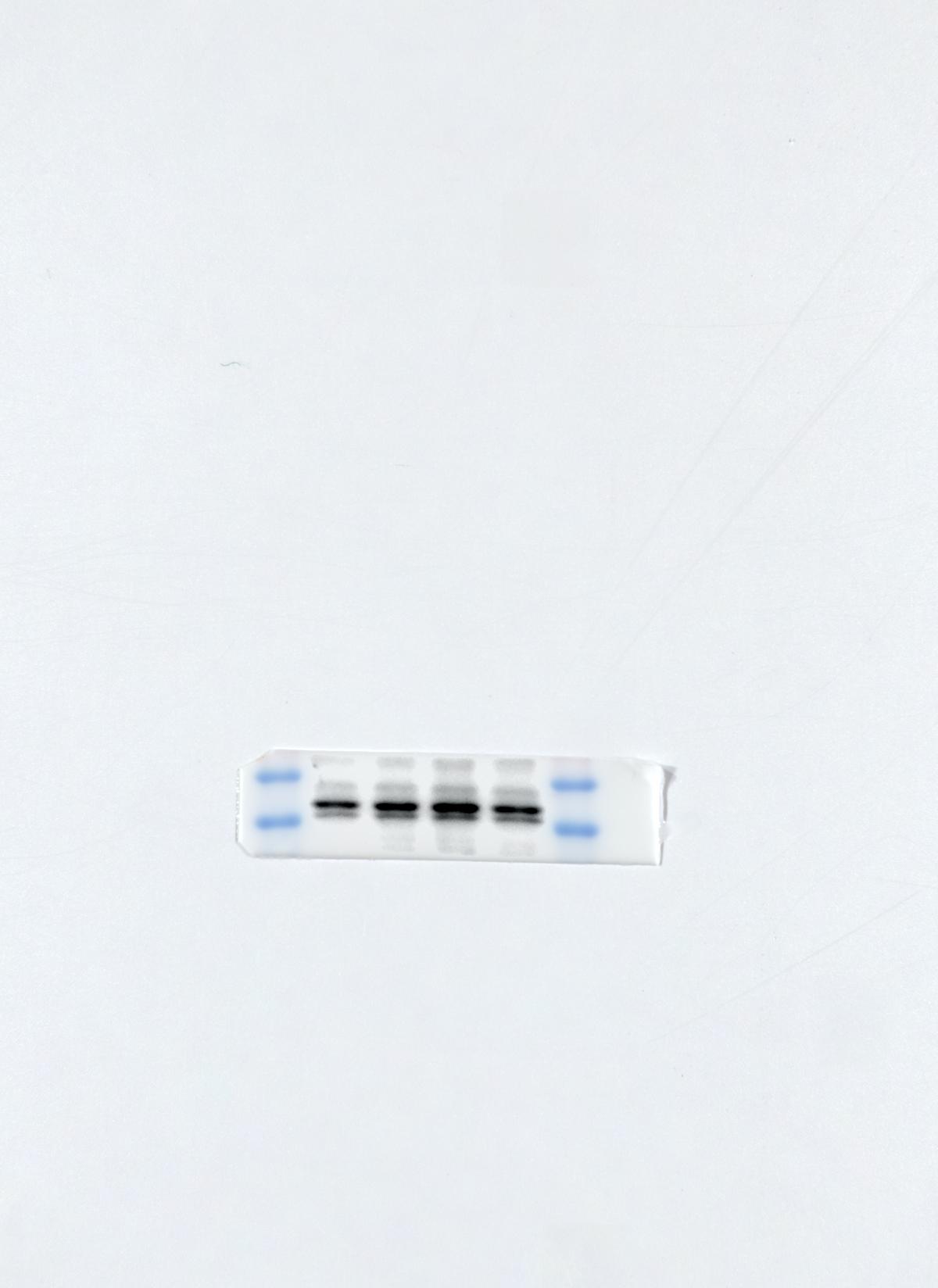
 β-Actin
